# Determinants of bone mass in older adults with normal- and overweight derived from the crosstalk with muscle and adipose tissue

**DOI:** 10.1038/s41598-023-31642-4

**Published:** 2023-03-28

**Authors:** Carina O. Walowski, Catrin Herpich, Janna Enderle, Wiebke Braun, Marcus Both, Mario Hasler, Manfred J. Müller, Kristina Norman, Anja Bosy-Westphal

**Affiliations:** 1 Institute for Human Nutrition and Food Science, Christian-Albrechts-University, Düsternbrooker Weg 17, 24105 Kiel, Germany; 2 Institute of Nutritional Science, University of Potsdam, Potsdam, Germany; 3 Department of Geriatrics and Medical Gerontology, Charité Universitätsmedizin Berlin, Corporate Member of Freie Universität Berlin and Humboldt-Universität zu Berlin, Berlin, Germany; 4 Department of Nutrition and Gerontology, German Institute of Human Nutrition, Potsdam-Rehbrücke, Nuthetal, Germany; 5 Department of Radiology and Neuroradiology, University Medical Center Schleswig-Holstein, Campus Kiel, Germany; 6 Applied Statistics, Faculty of Agricultural and Nutritional Sciences, Christian-Albrechts-University, Kiel, Germany; 7 German Center for Cardiovascular Research (DZHK), Partner Site Berlin, Berlin, Germany

**Keywords:** Ageing, Bone, Bone quality and biomechanics

## Abstract

Lower bone mass in older adults may be mediated by the endocrine crosstalk between muscle, adipose tissue and bone. In 150 community-dwelling adults (59–86 years, BMI 17–37 kg/m^2^; 58.7% female), skeletal muscle mass index, adipose tissue and fat mass index (FMI) were determined. Levels of myokines, adipokines, osteokines, inflammation markers and insulin were measured as potential determinants of bone mineral content (BMC) and density (BMD). FMI was negatively associated with BMC and BMD after adjustment for mechanical loading effects of body weight (r-values between −0.37 and −0.71, all *p* < 0.05). Higher FMI was associated with higher leptin levels in both sexes, with higher hsCRP in women and with lower adiponectin levels in men. In addition to weight and FMI, sclerostin, osteocalcin, leptin × sex and adiponectin were independent predictors of BMC in a stepwise multiple regression analysis. Muscle mass, but not myokines, showed positive correlations with bone parameters that were weakened after adjusting for body weight (r-values between 0.27 and 0.58, all *p* < 0.01). Whereas the anabolic effect of muscle mass on bone in older adults may be partly explained by mechanical loading, the adverse effect of obesity on bone is possibly mediated by low-grade inflammation, higher leptin and lower adiponectin levels.

## Introduction

Aging is characterized by the progressive decline of bone mass that is responsible for adverse outcomes like fracture risk and mortality^1^. Numerous studies indicate that a higher skeletal muscle mass is associated with a higher bone mineral density (BMD) in older adults^2–4^ whereas sarcopenia^5^ and high levels of fat mass (FM,^6–8^) exert a negative impact on bone. Therefore, the age-related decrease in muscle mass and increase in FM as well as fat infiltration in the musculoskeletal system may contribute to the impairment of bone mass leading to an obese osteosarcopenic phenotype and resulting in poorer overall strength and functionality^9^. As an underlying mechanism, the endocrine and paracrine crosstalk between bone and muscle or adipose tissue is suggested (for reviews see^10–12^). Due to the clinical importance of age-related musculoskeletal diseases, this ‘bone-muscle-fat crosstalk’ may reveal new targets to prevent or mitigate bone degradation.

The role of adipokines in the regulation of bone mass in older adults remains controversial. A pro-osteogenic effect has been shown for adiponectin that was found to promote osteoblastogenesis and inhibit osteoclastogenesis in in *vitro* and in *vivo* models^13,14^ whereas results from studies in older people often demonstrate a negative relationship between adiponectin and bone^15,16^. Likewise, leptin was reported to be positively as well as negatively associated with bone parameters in humans^17^. Accordingly, both osteogenic and osteolytic effects of leptin have been found in cell and animal models (for a review see^18^). Myokines, like irisin and myostatin, could be useful markers for the assessment of disorders of the muscle-bone unit and metabolic bone diseases or even therapeutic targets for the treatment of sarcopenia and osteoporosis. Irisin was found to be positively associated with bone mineral content (BMC)^19^ and negatively with the prevalence of fracture risk^19,20^*.* By contrast, myostatin negatively regulates bone mineralization while concurrently enhancing bone resorption by inhibiting osteoblast differentiation and promoting osteoclast differentiation^21,22^.

Vice versa, osteokines may exert anabolic or catabolic effects on muscle and adipose tissue (for reviews see^10–12^). Sclerostin, a negative regulator of bone growth secreted by osteocytes (for a review see^23^), has been shown to inhibit myogenesis in *vitro* and ex vivo^24^ and was observed to be negatively associated with muscle mass in humans^25^. Sclerostin is also thought to increase FM by promoting adipogenesis and lipid accumulation in pre-adipocyte cell lines^26,27^, in primary mesenchymal stromal cells from mice and humans^26^ and in animal experiments^28^. These findings are supported by positive correlations between sclerostin and FM in some^29,30^, but not all clinical trials^31^. In a recent study, sclerostin was identified as a putative new myokine that was found to impair the functional maturation of osteoblasts^32^. By contrast, osteocalcin, an osteoblast-derived marker of bone formation (for a review see^23^), has been shown to exert positive effects on muscle in *vitro*^33^, in *vivo*^34^ and in humans^35,36^ and to protect from obesity in men^37^ and women^38^.

These findings indicate that mechanistic experiments in *vitro* and in *vivo* do not always agree with results from human studies. Discrepant findings may be due to confounding effects of ageing associated diseases like metabolic impairment (i.e. insulin resistance, chronic inflammation) or decreased kidney function. The aim of the present study was therefore to identify potential determinants of bone mass and bone density derived from the crosstalk with skeletal muscle and adipose tissue in community-dwelling older adults in general good health with a wide BMI-range.

## Subjects and methods

### Study population

Data of 150 Caucasian men and women were collected at the ‘German Reference Center for Body Composition’ (Institute of Human Nutrition and Food Science at the University of Kiel, Germany) between 2019 and 2020 as described in detail elsewhere^39^. The primary aim of the study was to develop prediction equations for two seca medical bioelectrical impedance analysis devices for older adults. The trial was registered at clinicaltrials.gov as NCT04028648. Exclusion criteria were edema, chronic diseases, heart failure, renal failure, paralysis (e.g. after a stroke), neurodegenerative diseases, tumors in treatment, amputation of limbs, electrical and metallic implants, current alcohol abuse, not removeable piercings and large tattoos on the arms or legs (because of possible interference with magnetic resonance imaging (MRI) examinations) as well as medication which could influence body composition. The recruitment was realized using local advertisements and notice board postings. The study protocol was authorized by the medical ethic committee of the Christian-Albrechts-University of Kiel, Germany, and conducted according to the guidelines laid down in the ‘Declaration of Helsinki’. Written informed consent was received from each subject before participation^39^.

### Body composition analysis

Body weight was measured with subjects in underwear to the nearest 0.01 kg by an electronic scale (TANITA, Tokyo, Japan) connected to the BOD POD Body Composition System (COSMED SRL, Rome, Italy). Height was assessed without shoes using a stadiometer (SECA, Modell 285, Hamburg, Germany). Air-displacement plethysmography (BOD POD COSMED SRL, Rome, Italy) was performed to determine FM and fat-free mass (FFM) as previously described^40^. Absolute FM (kg) was calculated from body density using the equation by Siri et al.^41^. FFM (kg) was then calculated as the difference between body weight and absolute FM. FM-Index (FMI) and FFM-Index (FFMI) were calculated as FM (kg)/height (m^2^) and FFM (kg)/height (m^2^).

Measurements of skeletal muscle mass, subcutaneous and visceral adipose tissue (SAT and VAT) were performed using whole body MRI with a 1.5 T scanner (MAGNETOM Avanto, SIEMENS MEDICAL SYSTEMS, Erlangen, Germany)^42,43^. Subjects were examined in a supine position with arms extended above their heads and were required to hold their breath during scans in abdominal and thoracic regions. The whole body was scanned from wrist to ankle using continuous axial images of 8 mm slice thickness and 2 mm interslice gaps for arms, legs and trunk. Images were obtained using a T1-weighted-gradient echo sequence (repetition time: 157 ms; echo time: 4 ms for scans of arms, legs and abdominal region). Volumes of skeletal muscle mass, SAT and VAT were manually determined by using segmentation software (SLICEOMATIC 4.3, TOMOVISON, Montreal, Canada). VAT was defined as intra-abdominal fat between the top of the liver and femur heads. Volumes of total skeletal muscle (excluding head and neck muscles, hands and feet), VAT and SAT were determined from the sum of tissue areas (cm^2^) multiplied by the slice thickness. Volume data were then converted into tissue masses using the assumed densities of 1.04 g cm^−3^ for muscle and 0.92 g cm^−3^ for SAT and VAT^44^. Skeletal muscle mass was normalized to height squared using skeletal muscle mass index (SMI, (kg)/height (m^2^)).

Whole body BMC, BMD and T-Score were measured by dual X-ray absorptiometry (HOLOGIC Discovery A (S/N 82686), Inc., Bedford, MA, USA). Before daily measurements, a spine phantom calibration was performed. Manufacturer’s software (version 12.6.1:3, HOLOGIC, Inc.) was used for analysis. Results were summed up for both arms and legs, left and right ribs, thoracic and lumbar spine, pelvis as well as the head.

Hand grip strength (HGS) was measured using a hydraulic SAEHAN handgrip dynamometer (SH5001, Masan, South Korea). Subjects conducted the test in a standing position. The elbows were flexed at 90 degrees with the shoulder attached to the torso. HGS of the left and right hand was determined three times and the greatest value of the dominant hand was included in the analysis. The dominant hand was determined by self-disclosure.

### Endocrine parameters

After a minimum 10-h overnight fast, serum and plasma blood samples were taken from an antecubital vein and analysed as in detail described elsewhere^39^. Briefly, the participants were instructed to refrain from vigorous exercise and alcohol intake on the day prior to blood sampling*.* Serum was stored at room temperature in an upright position for 30 min for complete coagulation. Plasma and serum were obtained by centrifugation at 2000 g for 10 min at 20 °C and stored at −40 °C. Blood sample analyses were performed at the ‘German Institute of Human Nutrition’, Potsdam-Rehbrücke, Department of Nutrition and Gerontology, Nuthetal, Germany and a laboratory in Kiel, Germany^39^.

To investigate the interaction between and within bone, muscle and adipose tissue and its effects on body composition in advanced age, numerous humoral cytokines and growth factors were measured via commercial ELISA kits: the osteokines sclerostin (intra-assay CV: ≤ 7%, inter-assay CV: ≤ 10%; BIOMEDICA, Vienna, Austria) and osteocalcin (*n* = 93; intra-assay CV: 3.0–4.6%, inter-assay CV: 3.4–5.5%; BIOVENDOR, Brno, Czech Republic), the myokines myostatin (intra-assay CV: 1.8–5.4%, inter-assay CV: 3.1–6%; BIO-TECHNE, NE, Minneapolis, MN, USA) and irisin (intra-assay CV: 4.9–8.2%, inter-assay CV: 8.0–9.7%; BIOVENDOR, Brno, Czech Republic) and the adipokines leptin (intra-assay CV: 4.2–7.6%, inter-assay CV: 4.4–6.7%; BIOVENDOR, Brno, Czech Republic) and adiponectin (intra-assay CV: 2.8–3.9%, inter-assay CV: 5.9–6.4%; Immundiagnostik AG, Bensheim, Germany). As growth factor insulin-like growth factor 1 (IGF-1) (intra-assay CV: 5.1–6.7%, inter-assay CV: 5.5–6.6%; BIOVENDOR, Brno, Czech Republic) was measured. The inflammation markers interleukin 6 (IL-6) (intra-assay CV: 4.2–5.1%, inter-assay CV: 4.7–5.0%; BIOVENDOR, Brno, Czech Republic) and high-sensitivity C-reactive protein (hsCRP) (intra-assay CV: 0.73–5.73%, inter-assay CV: 1.50–5.76%; BECKMAN COULTER, Brea, CA, USA) were determined via commercial ELISA kit and immuno-turbidimetric test, respectively. Levels of insulin were measured by a chemiluminescent microparticle immunoassay (intra-assay CV: 1.4–2.1%, inter-assay CV: 1.5–2.2%; ABBOTT, Wiesbaden, Germany). Elevated insulin levels were set at > 25.0 mU/l and inflammation was based on hsCRP > 3 mg/l^39^.

### Statistical analysis

Statistical analyses were carried out with SPSS statistical software (SPSS 28.0, Inc., Chicago, IL, USA). All data are presented as means ± SD. Differences between independent samples were tested by unpaired t-test. Shapiro–Wilk test and residual analysis were used to verify normality^39^. Pearson's and Spearman's correlation coefficients were calculated to identify bivariate associations between and within body composition, hormones, growth factors, inflammation markers and HGS. Partial correlations were used to adjust for various confounders. Stepwise regression analyses were performed to access factors independently associated with BMC, BMD and T-Score. The qualitative factor sex (male or female) was coded numerically (1 or 2). All tests were two-sided and the level of significance was set at *p* < 0.05.

## Results

From the included 150 participants, data of 117 adults (71 women and 46 men) aged 60–82 years with a BMI between 18 and 37 kg/m^2^ were analysed. According to WHO criteria, the prevalence of overweight was 31.4% in women and 54.3% in men whereas 8.6% of women and 19.6% of men were obese. Data from 18 participants were excluded due to creatinine levels and estimated glomerular filtration rates exceeding the reference range. Further data from 15 subjects were excluded because of motion artefacts or incorrect patient positioning in MRI. Descriptive characteristics of the study population are summarized in Table 1.

**Table 1:** Characteristics of the study population.

	all subjects	women	men
*n*	117	71	46
age (y)	70.2 ± 5.0	69.6 ± 4.7	71.2 ± 5.8
height (m)	1.68 ± 0.10	1.62 ± 0.06***	1.77 ± 0.1
weight (kg)	73.1 ± 16.1	64.4 ± 10.5***	86.6 ± 13.6
BMI (kg/m^2^)	25.6 ± 3.9	24.4 ± 3.7***	27.4 ± 3.5
FMI (kg/m^2^)	9.3 ± 3.2	9.8 ± 3.3*	8.5 ± 2.8
SAT (kg)	17.5 ± 6.2	18.3 ± 6.2	16.3 ± 6.1
VAT (kg)	2.1 ± 1.5	1.4 ± 1.0***	3.2 ± 1.6
FFMI (kg/m^2^)	16.3 ± 2.4	14.7 ± 1.0***	18.8 ± 1.4
Skeletal muscle mass (kg)	22.6 ± 6.1	18.4 ± 2.6***	29.0 ± 3.7
SMI (kg/m^2^)	7.8 ± 1.4	7.0 ± 0.8***	9.2 ± 1.0
Bone - whole body			
BMC (kg)	2.05 ± 0.53	1.70 ± 0.28***	2.58 ± 0.36
BMD (g/cm^2^)	1.0 ± 0.1	0.9 ± 0.1***	1.1 ± 0.1
T-score	− 1.6 ± 1.2	− 2.1 ± 1.1***	− 0.8 ± 1.0
Bone - arms			
BMC (kg)	0.32 ± 0.11	0.24 ± 0.05***	0.44 ± 0.07
BMD (g/cm^2^)	1.4 ± 0.2	1.2 ± 0.1***	1.6 ± 0.1
Bone - legs			
BMC (kg)	0.83 ± 0.23	0.68 ± 0.12***	1.07 ± 0.16
BMD (g/cm^2^)	2.2 ± 0.3	2.0 ± 0.2***	2.5 ± 0.2
Bone - trunk			
BMC (kg)	0.53 ± 0.15	0.44 ± 0.08***	0.67 ± 0.12
BMD (g/cm^2^)	4.1 ± 0.6	3.8 ± 0.4***	4.5 ± 0.6
Bone trunk + bone legs			
BMC (kg)	1.37 ± 0.37	1.11 ± 0.18***	1.74 ± 0.26
BMD (g/cm^2^)	6.3 ± 0.9	5.7 ± 0.6***	7.1 ± 0.8
HGS (kg)	31.4 ± 10.5	24.7 ± 5.0***	41.8 ± 8.1

Values are means ± SD. BMI, body mass index; FMI, fat mass index; SAT, subcutaneous adipose tissue; VAT, visceral adipose tissue; FFMI, fat-free mass index; SMI, skeletal muscle mass index; BMC, bone mineral content; BMD, bone mineral density; HGS, hand grip strength. *p < 0.05, ***p < 0.001 sex differences by *t*-test. Bone parameters were not adjusted for body weight.

Men had a higher BMI, VAT, FFMI, skeletal muscle mass, SMI, BMC, BMD, T-Score (for whole body, arms, legs, trunk and the sum of trunk and legs) and HGS and a lower FMI compared with women.

Levels of hormones, growth factors and inflammation markers of the study population are summarized in Table 2.

**Table 2:** Levels of hormones, growth factors and inflammation markers in the total population and in the subgroups of men and women.

	all subjects	women	men
*n*	117	71	46
Insulin (µU/ml)	9.8 ± 6.7	8.7 ± 4.5*	11.6 ± 8.9
Leptin (ng/ml)	11.9 ± 11.0	14.5 ± 12.6***	8.0 ± 6.2
Adiponectin (mg/l)	18.8 ± 14.1	21.3 ± 16.0**	15.0 ± 9.5
IL-6 (pg/ml)	10.38 ± 26.06	6.09 ± 4.88*	17.00 ± 40.50
hsCRP (mg/l)	2.28 ± 3.30	2.09 ± 2.81	2.58 ± 3.97
Myostatin (ng/ml)	2.2 ± 0.8	2.2 ± 0.8	2.4 ± 0.8
Irisin (µg/ml)	6.6 ± 4.7	6.7 ± 4.7	6.5 ± 4.7
Sclerostin (pmol/l)	48.3 ± 23.2	43.0 ± 14.6**	56.5 ± 30.8
Osteocalcin (ng/ml)	14.8 ± 5.4	16.0 ± 5.4**	12.9 ± 4.9
IGF-1 (µg/l)	163.9 ± 57.8	147.4 ± 52.2***	189.2 ± 57.3

Values are means ± SD.

*IL-6*, interleukin 6; *hsCRP*, high-sensitivity C-reactive protein; *IGF-1*, insulin-like growth factor 1.

*p < 0.05, **p < 0.01, ***p < 0.001 sex differences by *t*-test.

Insulin, IL-6, IGF-1 and sclerostin levels were higher and leptin, adiponectin as well as osteocalcin levels were lower in men compared with women. The prevalence for elevated hsCRP levels in women and men with normal weight was 14.3% and 25.0% with respective values of 18.2% and 16.0% in the overweight and 66.7% and 11.1% in the obese group. By contrast, hyperinsulinemia was found in only 4% of men with obesity.

### Effects of muscle on bone parameters

In the total population, muscle mass and SMI were positively associated with bone parameters (for muscle mass, kg: BMC r = 0.82, BMD r = 0.63, T-Score r = 0.49, all *p* < 0.001 and for SMI, kg/m^2^: BMC r = 0.67, BMD r = 0.55, T-Score r = 0.41, all *p* < 0.001). In the subgroup of women, muscle mass was only positively correlated with BMC (r = 0.49,* p* < 0.001) whereas in men, muscle mass was associated with higher BMC, BMD and T-Score (BMC r = 0.53, BMD r = 0.37, T-Score r = 0.37,* p* < 0.01–*p* < 0.001). In the total population, the positive associations between skeletal muscle mass or SMI and bone parameters persisted after adjustment for weight (except for the correlation between SMI and T-Score) but were slightly weakened (for muscle mass, kg: BMC r = 0.58, BMD r = 0.48, T-Score r = 0.32, all *p* < 0.001 and for SMI, kg/m^2^: BMC r = 0.27, BMD r = 0.28, all *p* < 0.01). In the subgroups of men and women, the correlations between muscle mass and bone parameters remained no longer significant after adjusting for body weight. No relationships were found between myostatin or irisin and muscle mass, SMI or bone parameters. The results of our data did not change when instead of whole body parameters only the sum of the trunk and legs BMC or BMD was analysed.

### Effects of adipose tissue on bone parameters

After adjustment for body weight, FMI showed consistent negative associations with BMC, BMD and T-Score ranging between −0.37 and −0.71 in men and women (all *p* < 0.01). Concerning different fat compartments, VAT was negatively correlated with bone parameters in men when controlling for weight (BMC r = −0.43, *p* < 0.01, BMD r = −0.33, *p* < 0.05). Negative relationships were also observed between SAT and bone parameters after accounting for weight (men: BMC r = −0.52*, p* < 0.01, BMD r = −0.35, *p* < 0.05, T-Score r = −0.42, *p* < 0.01, women: BMC r = −0.32*, p* < 0.001).

Body fat compartments (SAT and VAT) and FMI were positively associated with leptin concentrations in both sexes and with inflammation markers in women whereas FMI was negatively correlated with adiponectin levels in men (Table 3).

**Table 3:** Correlation coefficients between BMI or fat compartments and inflammation markers, leptin or adiponectin in the subgroups of men and women.

	women				men			
	hsCRP	IL-6	Leptin	Adiponectin	hsCRP	IL-6	Leptin	Adiponectin
BMI	0.55**	0.20	0.77**	0.17	0.20	0.04	0.76**	− 0.31*
FMI	0.57**	0.20	0.80**	0.16	0.28	0.16	0.78**	− 0.29*
VAT	0.54**	0.25*	0.62**	0.03	0.25	0.06	0.74**	− 0.19
SAT	0.54**	0.25*	0.77**	0.17	0.23	0.06	0.70**	− 0.24

*hsCRP*, high-sensitivity C-reactive protein; *IL-6*, interleukin 6; BMI, body mass index; FMI, fat mass index; VAT, visceral adipose tissue; SAT, subcutaneous adipose tissue.

*p < 0.05, **p < 0.01.

IL-6 levels were negatively associated with bone parameters in men (BMC r = –0.34, BMD r = −0.34, T-Score r = −0.32, all *p* < 0.05, data are not adjusted for weight). Correlations remained significant after excluding two subjects with elevated IL-6 levels of 146 and 243 pg/ml. No relationship was observed between hsCRP or leptin and bone parameters. To test if an effect of hsCRP or leptin on bone could be masked by weight, partial correlation analyses adjusted for weight between hsCRP or leptin and bone parameters were performed and a significant negative association between leptin and BMC was found in men (r = −0.40, *p* < 0.01). Regarding inflammation markers, leptin levels were positively correlated with hsCRP (men: r = 0.36, *p* < 0.05, women: r = 0.54, *p* < 0.001) and with levels of IL-6 in women (r = 0.33, *p* < 0.01)*.* Leptin showed consistent positive correlations with skeletal muscle mass and SMI (ranging between 0.38 and 0.44 in men and women (all *p* < 0.01)).

### Effects of bone on bone parameters

Sclerostin levels were positively associated with bone parameters in the total population and in men whereas in women only a positive correlation between sclerostin and BMC was found (Table 4).

**Table 4:** Correlation coefficients between sclerostin or osteocalcin levels and bone parameters.

	all subjects		women		men	
	Sclerostin	Osteocalcin	Sclerostin	Osteocalcin	Sclerostin	Osteocalcin
BMC (kg)	0.38**	− 0.31**	0.31**	− 0.15	0.38**	− 0.12
BMD (g/cm^2^)	0.36**	− 0.32**	0.23	− 0.20	0.43**	− 0.22
T-score	0.36**	− 0.28**	0.23	− 0.30**	0.43**	− 0.20

Bone parameters were not adjusted for body weight.

BMC, bone mineral content; BMD, bone mineral density.

**p < 0.01.

Relationships between sclerostin and bone parameters remained significant after accounting for body weight or SMI as potential confounders in partial correlation analyses. In contrast to sclerostin, higher osteocalcin levels were associated with lower bone parameters in the total population and higher osteocalcin concentrations correlated with a lower T-Score in women. A negative association was observed between osteocalcin and sclerostin levels (all: r = −0.25, *p* < 0.05, men: r = −0.49, *p* < 0.01). The correlations remained significant after adjusting for weight.

IGF-1 levels were positively correlated with BMC, BMD and T-Score in the total population (BMC r = 0.33, BMD r = 0.37, T-Score r = 0.33, all* p* < 0.001). The positive association between IGF-1 and bone parameters persisted after adjustment for weight but was slightly weakened (BMC r = 0.21, BMD r = 0.23, T-Score r = 0.24, all *p* < 0.05).

Multiple stepwise regression analyses with BMC, BMD or T-Score as dependent variables and weight, FMI, SMI, leptin, leptin × sex, adiponectin, sclerostin, osteocalcin, IL-6, hsCRP, IGF-1, age and sex as independent variables were performed (Table 5).

**Table 5:** Stepwise multiple regression analyses with BMC, BMD and T-score as dependent variables.

Dependent variables and predictors	β coefficient	R^2^	SEE	*p*-value	VIF
**BMC** (kg)					
Model^a^					
weight	32.644	0.526	1.413	< 0.001	1.618
FMI	− 128.564	0.833	9.795	< 0.001	2.681
Sclerostin	2.608	0.864	0.877	0.004	1.239
Osteocalcin	− 13.799	0.875	3.569	< 0.001	1.223
Leptin × sex	4.040	0.890	1.197	0.001	2.142
Adiponectin	2.764	0.896	1.199	0.024	1.079
**BMD **(g/cm^2^)					
Model^a^					
sex	− 0.156	0.409	0.021	< 0.001	1.056
Sclerostin	0.001	0.458	0.000	0.006	1.056
**T-score**					
Model^a^					
sex	− 0.625	0.173	0.242	0.012	1.255
Sclerostin	0.017	0.240	0.005	0.001	1.101
IGF-1	0.005	0.286	0.002	0.019	1.200

BMC, bone mineral content; FMI, fat mass index; BMD, bone mineral content; *IGF-1*, insulin-like growth factor 1; SEE, standard error of estimation; VIF, variance inflation factor.

^a^Model: independent variables: weight, FMI, SMI, leptin, leptin × sex, adiponectin, sclerostin, osteocalcin, IL-6, hsCRP, IGF-1, age and sex.

Using BMC as dependent variable, the analysis revealed that weight and FMI were the main predictors, but the osteokines sclerostin and osteocalcin independently explained 4.2% of the variance and both adipokines leptin × sex and adiponectin further explained 2.1% of the variance. Concerning BMD and T-Score, sclerostin explained 4.7% or 6.7% of the variance in addition to the main predictor sex. As a further predictor for the T-Score, IGF-1 entered in the equation. The main predictors did not differ between the analyses of whole body BMC or BMD and the sum of trunk and legs BMC or BMD.

Regarding muscle, sclerostin levels were positively associated with SMI in the total population (r = 0.23, *p* < 0.05) and with HGS (women: r = 0.26, *p* < 0.05, all: r = 0.27, *p* < 0.01) whereas osteocalcin levels showed a negative correlation with SMI (all: r = −0.32, *p* < 0.01).

**Figure 1:**
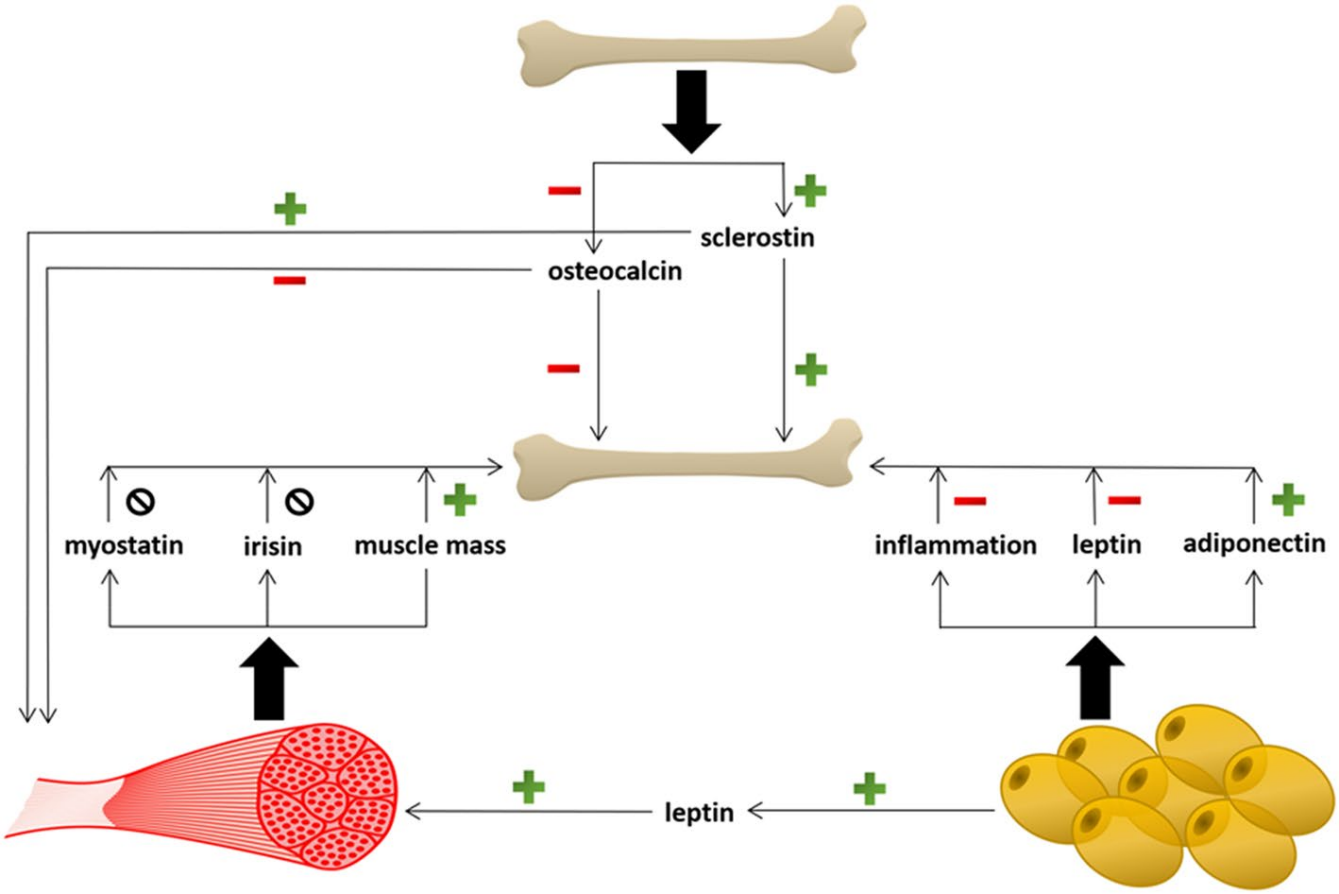
Overview of the regulation of bone mass or bone density.  positive correlation;  negative correlation;  no correlation.

In Fig. 1 an overview of the regulation of bone mass or bone density derived from the results of the present study is given.

## Discussion

### Crosstalk between bone and adipose tissue

The present data indicate that beside FMI both, subcutaneous and visceral obesity are strong predictors and critical risk factors of a low bone mass and density in older adults when controlling for the mechanical loading effects of total body weight on bone mass (see results). These results are confirmed by previous findings that identified FM^8,45–47^ or VAT^48–50^ as independent negative determinants of bone mass or bone density. The etiology of impaired bone health in obesity is multifactorial and a number of mechanistic explanations have been proposed to explain the inverse association between fat and bone (for a review see^51^). Beyond vitamin D deficiency, insulin resistance and reduced mobility, altered adipokine secretion and chronic pro-inflammatory status were identified as risk factors. An increased release of pro-inflammatory mediators has been shown to stimulate bone resorption (for a review see^52^)*.* In line with this mechanism, the present data demonstrated negative associations between IL-6 and BMC, BMD or T-Score in men (who had higher IL-6 levels compared to women, Table 2)*.* A higher expression of IL-6 mRNA has been found in bone samples from postmenopausal women with osteoporotic fractures compared to women with normal BMD^53^. Consistent with this clinical observation, knockout of the IL-6 gene in ovariectomized mice has been shown to prevent bone loss by upregulating mRNA expression of osteoblast-related genes and downregulating osteoclast-related mRNA^54^.

The negative association between FMI and BMC might also be explained by lower adiponectin levels with increasing FMI^55^ which we found in the subgroup of men (see results). Stepwise multiple regression analysis revealed that adiponectin was a significant positive predictor of BMC (Table 5). By contrast, previous human studies have demonstrated a negative relationship between adiponectin levels and BMD especially in advanced age^15,56^. The contradictory results may arise from distinct adiponectin concentrations or isoforms assessed by different ELISA kits as well as heterogenous study populations. In line with the findings of the present study, adiponectin has been demonstrated to promote proliferation, differentiation and mineralization in human osteoblasts^57^. With respect to the underlying mechanisms, transcription, translation and secretion of adiponectin as well as expression of its receptors were found in bone-forming cells^58^ and a pro-osteogenic role for adiponectin with increased osteoblastogenesis or lower osteoclastogenesis was found in in *vitro* and in *vivo* studies^13,59^.

Stepwise multiple regression analysis also revealed leptin × sex as a positive predictor of BMC (Table 5). Population based cross-sectional studies also found positive relationships between leptin and BMC and/or BMD adjusted for various confounders (e.g. age, %fat or BMI) in postmenopausal women^60–63^ and in men^63^ and reduced leptin levels in patients with vertebral fractures^61^. As an underlying mechanism, in *vitro* studies suggested that leptin exerts direct osteogenic effects mediated by its receptors in osteoblasts and osteoclasts^64–66^. Published data regarding the effects of leptin on bone parameters are however contradictory showing both anti-osteogenic as well as anabolic effects on bone formation (for reviews see^67,68^). In the present study, partial correlation analyses revealed a significant negative association between leptin and BMC in men after adjusting for weight (with BMD and T-Score showing a trend towards significance) confirming a sex-specific effect of this hormone. It has been suggested that leptin may exert diverging effects depending on whether central (via hypothalamus) or peripheral (via osteoblasts) mechanisms are operating^69–71^. The response of bone to leptin signaling might also differ between different skeletal sites (i.e. appendicular vs. axial) and bone structures (i.e. cortical vs. trabecular)^71–73^.

### Crosstalk between bone and muscle

The present data indicate positive effects of muscle mass on bone parameters in the total population. These findings are consistent with previously published results^3^. The bone-protective properties of muscle mass may be explained by increased weight bearing and mechanical effects due to muscle contraction whereas no relationships were found between myostatin or irisin and bone parameters (see results). In line with this supposition, the correlations between muscle mass and bone parameters weakened after adjusting for body weight (see results). In contrast to these findings, myostatin has been demonstrated as a negative regulator of bone in older subjects^74^ whereas irisin was shown to be associated with reduced risk of osteoporosis in postmenopausal women (for a review see^75^). Previous in *vitro* and in *vivo* studies have demonstrated higher myostatin concentrations in the context of a sedentary lifestyle^76^, obesity^77,78^ and pro-inflammatory environments^79^. Since the study sample comprises community-dwelling, mobile older adults in general good health, myostatin levels might be too low to exert a negative effect on bone tissue.

Higher skeletal muscle mass, kg and SMI, kg/m^2^ were correlated with higher leptin levels in men and women (see results). These findings are confirmed by various studies among older subjects^80–82^ and might be explained by an anabolic effect of leptin on muscle. In line with this supposition, in *vitro* experiments in cultured primary myoblasts have shown increased expression of myogenic genes by leptin treatment^83^ and an in *vivo* study in aged mice demonstrated that leptin administration increased the expression of microRNAs involved in myogenesis^84^.

### Autocrine effects on bone

The present data demonstrated positive associations between sclerostin and all bone parameters (Table 4). Multiple stepwise regression analyses also revealed that sclerostin was a positive predictor of BMC, BMD and T-Score (Table 5). These findings are in line with previous studies that reported a positive relationship between sclerostin and BMD in pre- and postmenopausal women as well as in men^29,85,86^. Further studies also demonstrated that postmenopausal women with osteoporosis exhibit lower levels of sclerostin than healthy controls^85,87,88^ and an increase in circulating sclerostin levels after risedronate treatment in patients with osteoporosis^87^. By contrast, levels of sclerostin have also been observed to be negatively associated with BMD in patients with hemodialysis^89^. However, end-stage renal disease impairs bone mass by other factors like secondary hyperparathyroidism. In recent mendelian randomization studies, evidence for bidirectional causal relationship between circulating sclerostin concentrations and BMD was proposed, with a positive effect of BMD on sclerostin levels, and a negative effect of sclerostin on BMD^90,91^. These findings suggest that the measurements of sclerostin may include both bioactive molecules and biomarkers of osteocyte activity (for reviews see^23,91^). Therefore, a reasonable explanation for the paradoxically positive association of sclerostin and bone parameters observed in our population may be due to the determination of total sclerostin. However, sclerostin is synthesized by osteocytes. Thus, the higher bone mass (i.e. more osteocytes), the higher is the overall synthesis and secretion of sclerostin.

In the present study, sclerostin levels were positively associated with SMI in the total population and with HGS in the total sample and in women. In agreement with these findings, higher serum sclerostin levels were shown to be associated with a lower risk of sarcopenia, low muscle mass and weak muscle strength in Korean older adults independent of age, sex and BMI^92^. In a recent study by Magarò et al.^32^, sclerostin was discovered in muscle cells in *vitro* and in muscles from variably aged mice suggesting sclerostin as a putative new myokine. Since muscle was positively correlated with bone parameters and sclerostin levels in the present study, the positive association between sclerostin and bone parameters might be explained by muscle tissue. Partial correlation analyses between sclerostin and BMC, BMD and T-Score adjusted for SMI and skeletal muscle mass however revealed, that the positive associations between sclerostin and bone parameters persisted.

Due to the effect of sclerostin in inhibiting bone formation, osteocalcin as a marker of bone formation, would be expected to be negatively correlated with sclerostin levels. This hypothesis is supported by the present data. The negative correlation between sclerostin and osteocalcin levels may therefore explain the unexpected negative relationship between osteoclacin and BMC, BMD and T-Score (Table 4). By contrast, multiple regression analyses revealed that osteocalcin was a negative predictor for BMC independent of sclerostin (Table 5). Negative effects of osteocalcin on bone health that have been proposed from studies in mice and in *vitro* experiments^93–95^ can therefore not be excluded.

### Strengths and limitations

The study population of older community-dwelling Caucasians was in good general health, with normal renal function, thus confounders caused by disease or relevant medication can be excluded. The population was also well characterized using whole body MRI which is considered as the gold standard method of the assessment of skeletal muscle mass. Nevertheless, the present findings should be considered in the context of some limitations. First, adiponectin circulates in blood in multiple (iso-)forms with different physiologic functions^96,97^. As only total concentrations were measured, the effects of the different (iso-)forms could not be examined. Second, since the primary aim of the study was to validate measures of bioelectrical impedance analysis vs. reference methods, lifestyle factors affecting bone mass were not assessed (e.g. smoking habits, sports, vitamin D supplementation). Third, the quality of the kits for the measurement of the various hormones might influence the results. Fourth, our study design was cross-sectional only. Thus, we could not address the possible skeletal response to endocrine and inflammatory determinants. In addition, skeletal response to an intervention is a function of bone architecture (trabecular and cortical) which was not differentiated by DXA-derived measures of BMC and BMD^98^. Finally, the findings need to be confirmed using a longitudinal study design.

In conclusion, in contrast to skeletal muscle mass, our results suggest that FMI and different body fat compartments are strongly related to lower bone mass and density in older adults and possibly mediated by low-grade inflammation, higher leptin and lower adiponectin levels. In line with other human studies and in contrast to animal and cell culture experiments the present study reveals sclerostin as an important positive predictor of bone mass and density. Further investigations are needed to clarify this paradox.

## Data Availability

The datasets generated during and/or analysed during the current study are available from the corresponding author on reasonable request.
